# INEAS’s Cost-Effectiveness Analysis of Vemurafenib: Paving the Way for Value-Based Pricing in Tunisia

**DOI:** 10.3390/jmahp12040023

**Published:** 2024-10-06

**Authors:** Mouna Jameleddine, Nabil Harzallah, Hela Grati, Marie Christine Odabachian Jebali, Jaafar Chemli, Sebastián García Martí, Natalie Soto, Andrés Pichon-Riviere, Chokri Hamouda

**Affiliations:** 1 The National Authority for Assessment and Accreditation in Healthcare (INEAS), Tunis 1002, Tunisia; n.harzallah@ineas.tn (N.H.);; 2 Institute for Clinical Effectiveness and Health Policy (IECS), Buenos Aires 1414, Argentina

**Keywords:** vemurafenib, advanced melanoma, targeted therapy, BRAF V600, cost-effectiveness, cost utility, health technology assessment, value-based pricing, Tunisia, market access

## Abstract

The Tunisian Health Technology Assessment (HTA) body, INEAS, conducted a cost-effectiveness analysis (CEA) of vemurafenib in the treatment of locally advanced or metastatic BRAF V600-mutated melanoma. The objective of this analysis was to enable the use of value-based pricing as a new approach to price negotiation. This study was part of a broader HTA report that was prepared in response to a joint request from the regulatory authorities and the CNAM, Tunisia’s compulsory insurance scheme. Our analysis was based on a probabilistic Markov cohort model that calculated the costs and quality-adjusted life years (QALY) associated with vemurafenib compared to the standard of care from a public payer perspective. The CEA indicated that vemurafenib provides a gain of 0.38 life years (1.78 vs. 1.4) for an incremental cost of USD 101,106.62 from the perspective of the main public payer (CNAM). This study revealed an incremental cost-effectiveness ratio (ICER) of 163,311.40 USD/QALY and 163,911.46 USD/QALY, respectively, from the CNAM and public health facilities’ perspectives. Vemurafenib cannot be considered cost-effective in terms of what has normally been considered a reasonable willingness to pay (WTP) in Tunisia. A significant price reduction would be necessary to bring the incremental cost-effectiveness ratio to an acceptable level.

## 1. Introduction

Melanoma, a type of skin cancer that originates from pigment-producing cells, is a major challenge in oncology, requiring advanced and innovative approaches for its effective management [1]. Due to its insidious nature, melanoma is often diagnosed at an advanced stage, resulting in a poor prognosis. Global statistics for 2020 indicate a significant burden, with 324,635 new cases worldwide, as reported by GLOBOCAN [2]. While surgical intervention stands as the cornerstone of therapeutic interventions at early stages, managing advanced melanoma, particularly in Stages IIIc and IV, represents a challenge to healthcare practitioners and systems alike. The prognosis for patients in these stages remains poor [3].

Prior to the advent of targeted therapies, including vemurafenib, dacarbazine-based chemotherapy was considered the standard of care for advanced melanoma [4]. Vemurafenib was the first selective BRAF inhibitor licensed in cancer treatment. It is indicated for the treatment of patients affected by advanced melanoma with BRAF V600 mutations, found in 50% of melanoma cases and associated with increased cell proliferation and increased oncogenic cell activity [5].

The National Authority for Assessment and Accreditation in Healthcare (INEAS), the Health Technology Assessment (HTA) body in Tunisia, conducted a de novo cost-effectiveness analysis of vemurafenib for the treatment of locally advanced or metastatic BRAF V600-mutated melanoma in Tunisia. This analysis was an essential part of a broader HTA report that was produced in response to a joint request of the regulatory authorities and the CNAM, Tunisia’s compulsory insurance scheme, to support potential coverage decision and price negotiations through value-based pricing (VBP) [6].

## 2. Materials and Methods

### 2.1. Selection of the Model Type and Structure

To identify a robust model with which to assess the cost-effectiveness of vemurafenib in patients with BRAF V600-mutated metastatic and/or unresectable melanoma, we conducted a comprehensive systematic review of pharmacoeconomic studies. Searches were conducted in Medline (via Pubmed), the Cochrane Library, and CRD (NH SEED). Documents were selected independently by two reviewers. In the case of multiple publications, only the most recent version was included. Disagreements were resolved by consensus. Overall, 34 references were identified through database searches. A hand search of specialized journals identified an additional 12 cost-effectiveness analyses that were not retrieved through the database searches. Overall, 44 records were included after duplicates were removed. After the examination of titles and abstracts, 37 references were excluded and 7 were read in full (5 original articles and 2 systematic reviews). The selected studies underwent an in-depth quality assessment using the Fichas de Lectura Crítica (FLC) 3.0 tool to ensure methodological consistency [7]. Finally, 5 studies were assessed for methodological quality using the FLC 3.0 tool. The search strategy and results are detailed in the Supplementary Materials Tables S1 and S2. A PRISMA flowchart illustrating the selection process is provided in Supplementary Materials Figure S1, while summaries of the selected studies are included in Supplementary Materials Tables S3 and S4 [3,4,8,9,10,11,12]. This approach enabled us to identify a validated model with demonstrable relevance to our research objectives.

### 2.2. Decision Model

**Figure 1:**
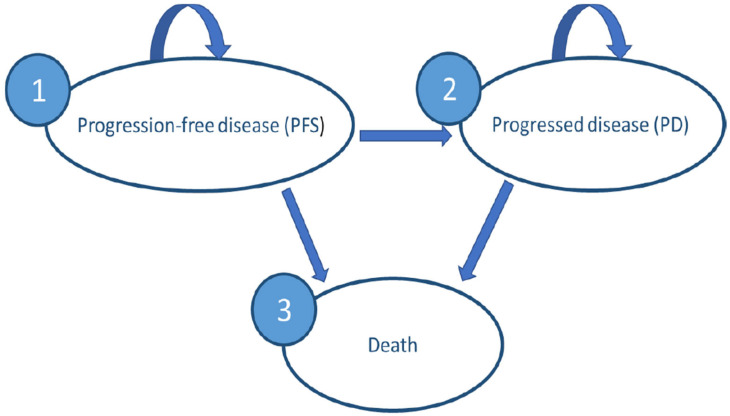
Markov model with three health states.

To assess the cost-effectiveness of vemurafenib for patients with BRAF V600-mutated metastatic and/or unresectable melanoma, we developed a probabilistic decision analytic model. The model compares two scenarios: treating all patients with vemurafenib (960 mg (4 × 240 mg tablets) twice daily) versus the status quo (dacarbazine, 300 mg/m^2^/day for 3 days every 3 weeks). All patients were over 18 years, treatment-naïve, and had BRAF V600 unresectable or metastatic melanoma. Both treatments were continued until disease progression or occurrence of unacceptable toxicity. We created a Markov model using Microsoft Excel 2016^®^ with three mutually exclusive disease-related health states (Figure 1): (1) progression-free survival (PFS), (2) progressed disease (PD), and (3) death. A time horizon of 10 years, with monthly cycles, was chosen after reviewing the input from the identified CEAs and consulting with experts in the field. The analysis was carried out from the perspective of Tunisian public payers (CNAM and public health facilities). Costs and health outcomes were discounted at 5% per year in accordance with INEAS guidelines [13]. A PFS state was the starting point for the simulated cohort of patients. In each cycle, a proportion of patients could remain at the same health state, another proportion could progress, and another may die, according to transition probabilities. Regression from the PD state to the PFS state was not possible.

#### 2.2.1. Model Parameters

- Transition probabilities

As a starting point, we modelled the actual scenario in Tunisia where all patients were treated with dacabarzine, assuming the same rate of progression in the Tunisian population as the one observed in the research of Robert C et al., 2011 [14]. The progression-free survival (PFS) and overall survival (OS) curves were digitized to obtain state occupancy times in the PFS and alive states, respectively. The progressed disease (PD) state time was derived from the difference between the other two values.

To estimate the effect of the new intervention, a comprehensive systematic search was carried out. Six records were identified, and two systematic reviews were selected for further analysis [3,15]. Both reviews were found to be of good quality following assessment using the FLC 3.0 tool [7]. Details of the search strategy, results, and evidence tables are provided in Supplementary Materials Tables S5–S7. A PRISMA flow chart, which illustrates the process of selecting studies for inclusion in the analysis, is presented in Figure S2. Hazard ratios (HRs) for OS and PFS were extracted from the most recent review (Franken et al., 2019) and then applied to the cycle hazards of the corresponding health states of the dacarbazine cohort [15]. These new hazards were then used to estimate the survival probability using the formulas available in the research of Briggs et al., 2011 [16].

- Health-related quality of life

A preliminary literature search revealed a lack of health-related quality of life (HRQOL) data for melanoma patients in Tunisia. A systematic search of the bibliographic databases Medline (via Pubmed) and Web of Science, as detailed in Supplementary Materials Table S8, was conducted to identify relevant utility values. We only included systematic reviews in English or French that reported health-related quality-of-life outcomes in the adult population with advanced melanoma treated with chemotherapy (dacarbazine) or targeted therapy, using direct or indirect assessment methods. Publications were independently reviewed by two researchers. Duplicates were eliminated and, in the case of multiple publications, only the most recent version was retained. A PRISMA flow chart illustrating the publication selection process is shown in Supplementary Materials Figure S3. The key resource identified in the process was outlined in the research of Tran et al., 2019 [17]. The evidence table can be found in Supplementary Materials Table S9.

- Costs

Only direct medical costs were considered in our analysis, including those of drug administration and acquisition (considering a 45% price cut on the initial price proposed by the manufacturer), patient follow-up in the various health states (medical visits, biological tests, diagnostic procedures, costs of severe adverse events, palliative care, and radiotherapy), and BRAF genetic mutation diagnoses performed at Institut Pasteur of Tunis

The costs of each strategy were estimated from the perspective of public payers, the National Health Insurance Fund (CNAM) and public health facilities (PHFs), in accordance with INEAS guidelines for cost-effectiveness analyses (CEA) [13]. The costs were calculated by multiplying the unit costs of each resource by their level of utilization in Tunisia for each health state. The rate of utilization was determined and validated by an expert group in accordance with clinical practices in the country.

○ The source of unit costs differs depending on the perspective: The CNAM Perspective

There are three types of CNAM reimbursement schemes in Tunisia: the public scheme, the third-party payment scheme, and the reimbursement scheme. Each scheme has different reimbursement methods and rates. The unit costs for the public scheme were calculated based on the 2018/2020 agreement between the Ministry of Health and the Ministry of Social Affairs for care services provided in public facilities [18,19]. For the other two schemes, the unit costs of services provided in private healthcare facilities were calculated based on the agreement between CNAM and private laboratories and practitioners, as well as on the 2006 legal text that established the general nomenclature for medical procedures by private sector practitioners [20,21]. To determine the final unit cost from the CNAM perspective, we calculated a weighted average based on the percentage of affiliates in each scheme. The public scheme accounted for 56%, while the other two schemes accounted for a total of 44% [22].

○ The public health facilities (PHFs) perspective

Unit costs were calculated on the basis of the PHFs tariffs for full-paying patients, as outlined in the 2008 decree of the Minister of Finance and the Minister of Health and the 2006 decree of the Minister of Health, setting the general nomenclature for medical procedures [23,24]. Only adverse events requiring hospitalization (grades 3 and 4) were considered. Monthly costs were then calculated by multiplying the probability per cycle of such an event by its average management cost. The probabilities of these adverse events occurring in patients treated with dacarbazine and vemurafenib were extracted from the research of Pike et al., 2017 [3] and Franken et al., 2019 [15], respectively.

#### 2.2.2. Validation of the Model

The face validity of the model was checked in collaboration with clinical experts. Internal validity was verified by a “walk-through” of the model with peers. Extreme value analysis was also performed. External validity was verified through comparison with the outcomes of clinical studies.

#### 2.2.3. Sensitivity Analyses

Given that the inputs of the model are subject to uncertainty, the impact of various parameters on the results has been analyzed. A one-way deterministic sensitivity analysis (DSA) was conducted to test the robustness of the model and identify the most influential parameters, as well as to evaluate their impact on the results. Table 1 presents the parameters that were subjected to a deterministic sensitivity analysis. The confidence interval limits for HRs were extracted from the meta-analysis conducted by Franken et al. (2019) [15], while those for utilities were derived from the research of Tran et al. 2018 [17]. A rate of ±20% was applied to the other parameters in accordance with INEAS guidelines [13].

A probabilistic sensitivity analysis (PSA) was conducted to assess the robustness of the analysis and to explore the consequences of uncertainties related to influential parameters. The PSA was performed using a Monte Carlo simulation, which involved randomly sampling over 1,000 iterations. The inputs and assumptions that were tested included HRs for OS and PFS (lognormal distribution) as well as utility estimations (beta distribution), aligning with established practices in health economic modeling and with the report of the ISPOR-SMDM Modeling Good Research Practices Task Force Working Group–6: [25]. Acceptability curves, evaluating different vemurafenib price-cut scenarios, were also developed.

## 3. Results

Table 2 and Table 3 provide details of the costs used as inputs for our model.

The key parameters of the model are summarized in Table 4.

The discounted results show a gain of 0.62 QALYs for an incremental cost of USD 101,474.12 from the public health facilities’ perspective and of USD 101,057.35 from the CNAM perspective. These results correspond to an ICER of 163,893.64 USD/QALYs and 266,553.31 USD/life years gained (LYGs) from a PHF perspective versus 163,238.60 USD/QALY (265,445.59 USD/LYG) from a CNAM perspective (Table 5). If the company covers the cost of BRAF testing, the ICER would be 162,787.06 USD/QALY from the PHF perspective and 162,298.31 USD/QALY from the CNAM perspective.

- Sensitivity analysis

**Figure 2:**
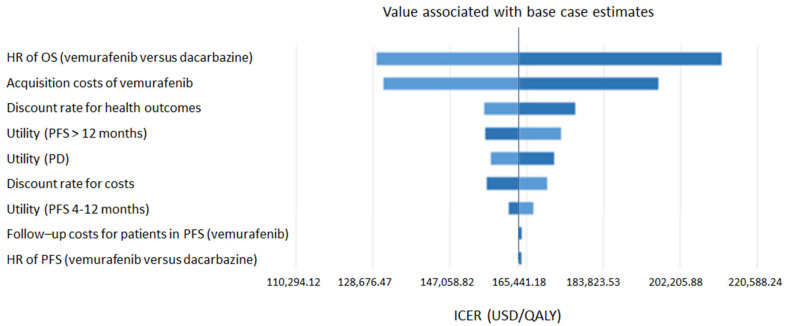
Tornado diagram illustrating the impact on the results of uncertainty in each parameter (CNAM perspective).

The deterministic sensitivity analysis, illustrated by the Tornado diagram (Figure 2), demonstrated that the most significant parameters influencing the results are the overall survival HR and the acquisition cost of vemurafenib in the two perspectives. Other parameters, such as utilities, patient follow-up costs, or the costs of managing adverse effects, had little impact on the results.

**Figure 3:**
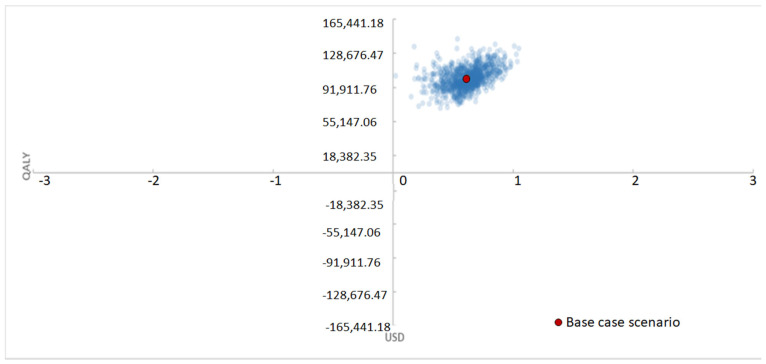
Probabilistic sensitivity analysis (PHFs perspective).

The results were found to be robust in all the tested scenarios (Figure 3).

**Figure 4:**
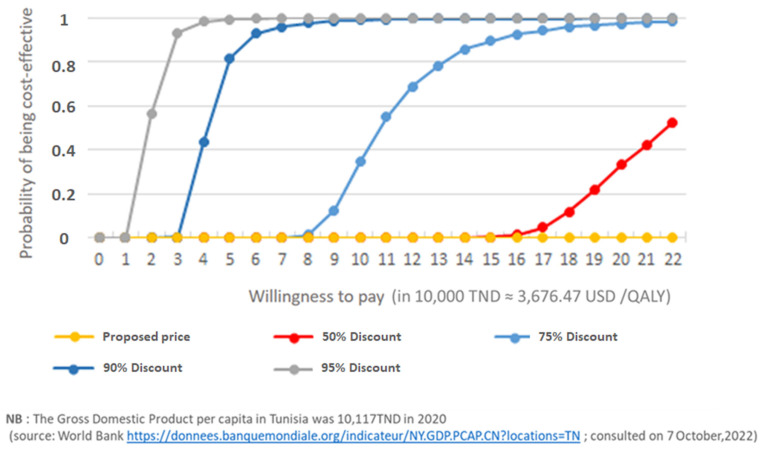
Acceptability curves.

Figure 4 illustrates the probability of the treatment being cost-effective according to the willingness to pay (expressed in 10,000 TND ≈ 3676.47 USD/QALY) for different scenarios of potential discounts on the proposed price. The probability of the product being cost-effective at the proposed price is 0%.

## 4. Discussion

Our analysis indicates that vemurafenib provides a gain of 0.38 life years (1.78 vs. 1.4) for an incremental cost of 101,057.35 USD from the main public payer perspective (CNAM). These results correspond to an ICER of 163,893.64 USD/QALYs (266,553.31 USD/LYG) from a PHF perspective and 163,238.60 USD/QALY (265,445.59 USD/LYG) from a CNAM perspective. According to sensitivity analyses, the most influential parameters on the results are the HR of overall survival and the acquisition cost of vemurafenib. Regarding the transition probabilities, we used the same progression observed in the research by Robert C et al., 2011 [14] adapted to the Tunisian population. It would have been better to have patient-level local data; however, this type of data is not common in many LMICs. The probability of the product being cost-effective at what is considered a reasonable willingness-to-pay threshold in Tunisia is 0% in all tested scenarios. A significant price reduction would be necessary to bring the incremental cost-effectiveness ratio to an acceptable value. These results are consistent with those of other CEAs conducted in various settings, including high-income countries [3,7,8,9,10]. The published analyses in the United States and Norway suggest that price reductions of 72% and 81% are needed to reach efficiency thresholds of $100,000/QALY and €55,850/QALY, respectively. This problem is common when new therapies are evaluated against old treatments and is even more critical in low- and middle-income countries, where resources are limited. INEAS has recommended that decision-makers negotiate the proposed price with the company based on the CEA results. The price recommended by INEAS to the decision-makers was not included in the HTA report in order to allow confidential negotiations with the company [6].

The issue of high drug prices is creating barriers to access and an increased economic burden for both patients and health insurance systems, particularly in resource-constrained contexts [26]. As a result, LMICs often struggle to incorporate established medications into their treatment protocols, despite their proven efficacy. In the case of advanced melanoma, the therapeutic arsenal is constantly evolving. At the time of this analysis, the only drug in the pipeline for approval in Tunisia was vemurafenib, a targeted therapy. In countries with higher incomes, the treatment of the disease has evolved to include immunotherapy or, for those ineligible for immunotherapy, targeted therapy with a combination of BRAF and MEK inhibitors rather than either inhibitor administered as a monotherapy. This approach offers an added survival benefit and reduced toxicity, making it a preferable choice. [27,28].

The pricing policies used for price control in most countries include international reference pricing (IRP) and value-based pricing (VBP). The WHO collaborating center for Pricing and Reimbursement Policies defines IRP as follows: “IRP as the practice of using the price(s) of a medicine in one or several countries in order to derive a benchmark or reference price for the purposes of setting or negotiating the price of the product in a given country” [29]. In contrast, VBP sets drug prices based on the value they provide in a given setting. The fundamental principle of the VBP approach is that the costs of drugs should not exceed their health benefits, and it is based on an implicit or explicit decision rule, which compares the incremental cost-effectiveness ratio of a drug with a willingness-to-pay threshold, e.g., £20,000 to £30,000 per QALY gained in England and Wales [30].

Our CEA is one of the first to be conducted by an HTA agency in the Middle East and Africa region with the aim of strengthening price negotiations through the use of value-based pricing, as outlined in the WHO guideline 2020 on country pharmaceutical pricing policies [31]. Value-based pricing through HTA represents a new concept in price negotiation in Tunisia, as the country has historically used IRP for pricing newly launched medicines like many other low- and middle-income countries (LMICs) [32]. However, these list prices are unreliable benchmarks due to confidential rebates [33]. As a result, IRP, initially used as a cost containment tool, may lead to higher relative prices in LMICs, undermining initiatives to improve the accessibility and affordability of innovative medicines.

The lack of affordable treatments will inevitably exacerbate the issue of judicialization of care, where individuals turn to legal courts to obtain necessary medications not readily available through traditional healthcare channels. This phenomenon is prevalent across LMICs and is a cause for concern in Tunisia due to its potential to exacerbate inefficiency and inequity. It may also pose a threat to the financial sustainability of health systems [34]. These challenges underscore the necessity for pricing models that reconcile affordability for health systems and patients with incentives for innovation, while guaranteeing fair access to life-saving medications. In addition, due to the monopsony position of the pharmaceutical industry, payers, including those in high-income countries, frequently encounter the dilemma of accepting inefficient prices or denying reimbursement to patients. This may also result in a delay in the entry of drugs to the market until biosimilars or generics become available.

## Figures and Tables

**Table 1:** Deterministic sensitivity analysis (CNAM perspective).

Parameter	Base Value	Range
Discount rate	5%	3–8%
HR PFS	0.38	0.32–0.45
HR OS	0.81	0.68–0.96
Probability of serious AEs related to dacarbazine	0.016	0.013–0.02
Probability of serious AEs related to vemurafenib	0.029	0.025–0.034
Utility PFS state (1 day–3 months)	0.69	0.665–0.715
Utility PFS state (4–12 months)	0.905	0.858–0.952
Utility PFS state (≥12 months)	0.910	0.863–0.957
Utility PD state	0.45	0.403–0.497
Dacarbazine administration costs (TND)	159.21	127.43–191.16
Vemurafenib acquisition costs (TND)	6178.76	4944.47–7407.68
Follow-up costs in PFS state (dacarbazine cohort)	65.11	52.00–78.04
Follow-up costs in PFS state (vemurafenib cohort)	63.06	50.40–75.54
Follow-up costs in PD state	42.79	34.19–51.23
Cost of managing grade 3–4 AEs of dacarbazine	423.89	338.97–507.70
Cost of managing grade 3–4 AEs of vemurafenib	423.89	338.97–507.70
Proportion of patients with BRAF mutation	0.5	0.4–0.6

**Table 2:** Follow-up costs in the progression-free survival state (USD).

Interventions	Utilization Rate per Cycle	Unit Cost (PHF)	Monthly Cost (PHF)	Unit Cost (CNAM/Public Scheme	Unit Cost (CNAM /Third-Party Payment and Reimbursement Schemes)	Monthly Cost CNAM (Weighted Average)
Administration costs of dacarbazine	4.34	17.65	76.63	Included in the daily fee *	44.12	173.72
Follow-up costs (dacarbazine cohort)						
Consultation with a specialist	1.45	5.15	7.46	Included in the daily fee *	16.54	23.95
Biological tests	1.45	20.00	28.94	Included in the daily fee *	34.93	22.316
Abdominal and pelvic computed tomography (CT) scan	0.33	88.24	29.41	73.53	115.81	30.68
Bone scan	0.008	49.63	0.411	66.18	66.18	0.55
Magnetic resonance imaging (MRI)	0.008	147.06	1.23	125.00	110.29	0.99
Follow-up costs (vemurafenib cohort)						
Consultation with a specialist	1	5.15	5.15	12.87	16.54	14.47
Biological tests	1	21.18	21.18	0	37.06	16.31
Abdominal and pelvic CT scan	0.33	88.24	29.41	73.53	115.81	30.68
Bone scan	0.008	49.63	0.41	66.18	66.18	0.55
MRI	0.008	147.06	1.23	125.00	110.29	0.99

* A daily fee of USD 36.76 includes the cost of administering the treatment, a visit to a specialist, and biological tests/biological analyses.

**Table 3:** Follow-up costs in the progressed disease state (TND).

Interventions	Monthly Rate	Unit Costs PHF	Monthly Cost PHF	Unit Cost CNAM Public Scheme	Unit Cost CNAM Third-Party Payment and the Reimbursement Schemes	Monthly Cost CNAM (Weighted Average)
Surgery	0.008	25.74	0.21	82.72	110.29	0.69
Outpatient palliative treatment	0.167	17.65	12.18	17.65	44.12	4.88
Palliative treatment requiring hospitalization	0.05	613.24	30.68	613.24	613.24	30.68
Radiotherapy	0.008	477.94	3.98	477.94	477.94	3.98

**Table 4:** Key parameters used in the base case analysis.

Definition	Value		Source/Note
Efficacy estimate for vemurafenib	Overall survival (95%CI)	Progression-free survival (95%CI)	Franken et al., 2019 [15]
	0.81 (0.68–0.96)	0.38 (0.32–0.45)	
Adverse events			
Probability of serious adverse events associated with dacarbazine per cycle	0.016		Pike et al., 2017 [3]
Relative risk of serious adverse events associated with vemurafenib compared to dacarbazine	1.75 (1.51–2.03)		Franken et al., 2019 [15]
Probability of serious adverse events associated with vemurafenib per cycle	0.0287		
Quality-adjusted life years (QALY) weight			
Utility (PFS)	0.69 (0–3 month) 0.905 (3–12 month) 0.910 (>12 month)		Tran et al., 2019 [17]
Utility (PD)	0.45		Tran et al., 2019 [17]
Costs per cycle			
	CNAM perspective (USD)	PHF perspective (USD)	
Acquisition cost of dacarbazine	152.43		Regulatory authorities
Acquisition cost of vemurafenib	6170.61		Regulatory authorities
Administration cost of dacarbazine	173.72	76.63	
Monitoring costs in PFS (vemurafenib cohort)	63.04	57.31	
Monitoring costs in PFS (Dacarbazine cohort)	65.09	67.47	
Monitoring costs in PD	40.25	37.78	
Costs associated with the management of serious adverse events (AEs) of vemurafenib	12.15		
Costs associated with the management of serious AEs of dacarbazine	6.94		
Cost of BRAF mutation testing	177.21		Institut Pasteur–Tunis

**Table 5:** Discounted incremental cost-effectiveness analysis results.

Intervention	Total Cost (USD)	Effectiveness (QALYs)	Effectiveness (LYG)	Incremental Cost	Incremental Effectiveness QALY	Incremental Effectiveness LYG	ICER USD/ QALY	ICER USD/ LYG
PHF perspective								
Dacarbazine	1865.90	0.76	1.4	NA	NA	NA	NA	NA
Vemurafenib	103,298.53	1.38	1.78	101,474.12	0.62	0.38	163,893.64	266,553.31
CNAM perspective								
Dacarbazine	2332.72	0.76	1.4	NA	NA	NA	NA	NA
Vemurafenib	103,418.90	1.38	1.78	101,057.35	0.62	0.38	163,238.60	265,445.59

## Data Availability

The raw data supporting the conclusions of this article will be made available by the authors on request.

## Supplementary Materials

The following supporting information can be downloaded at: https://www.mdpi.com/article/10.3390/jmahp12040023/s1, Table S1: Search strategy for pharmaco-economic studies; Table S2: Search results for pharmaco-economic studies on PubMed; Table S3: Overview of selected original pharmaco-economic studies; Table S4: Overview of systematic reviews of pharmaco-economic studies; Table S5: Search strategy for clinical inputs; Table S6: Search results for clinical inputs on PubMed; Table S7: Evidence tables of selected systematic reviews (extracted from FLC); Table S8: Search strategy for utilities in patients with advanced melanoma; Table S9: Evidence table of the selected studies on health-related quality of life (extracted from FLC); Figure S1: PRISMA flow chart for pharmaco-economic studies; Figure S2: PRISMA flow chart for clinical studies; Figure S3: PRISMA flow chart for studies on health-related quality of life.
