# Supplementary material for: INEAS’s Cost-Effectiveness Analysis of Vemurafenib: Paving the Way for Value-Based Pricing in Tunisia
Source: J Mark Access Health Policy. 2024 Oct 6;12(4):294–305. doi: 10.3390/jmahp12040023 (PMC11503406; doi:10.3390/jmahp12040023)
Supplement: Supplementary file 1 [file jmahp-12-00023-s001.zip › jmahp-2937027-supplementary.pdf]

Supplementary material

Table S1: Search strategy for pharmaco-economic studies

**Databases and journals**	
**Medline (via PubMed), Cochrane Library, CRD (NH SEED) databases and in two specialized journals Pharmacoeconomics and Value in Health**	
**Timeframe**	
**Date: January 2011 to December 2020**<br>**Update: Mars 2021**	
**Inclusion criteria**	
**Population**	Patients with unresectable or metastatic BRAF V600 mutated melanoma
**Intervention**	Vemurafenib
**Comparator**	Dacarbazine
**Outcomes**	Cost per QALY, cost per life-year gained
**Study design**	Cost-effectiveness, cost-utility
**Exclusion Criteria**	
**Language**	Other than english or French
**Type of publication**	Conference abstracts, grey literature
**Keywords**	
1. **Advanced melanoma**<br>2. **Vemurafenib**<br>3. **Targeted therapy**<br>4. **Dacrabazine**<br>5. **Chemotherapy**<br>6. **Cost effectiveness analysis**<br>7. **Cost utility analysis**<br>8. **Economic analysis**	

Table S2: Search results for pharmaco-economic studies on PubMed

Search #	Search equation	Number of documents (01/2011-12/2020)	Mars 2021
#1	Advanced melanoma	7,200	7,740
#2	Vemurafenib	2,433	2,521
#3	Targeted therapy	442,949	464,857
#4	Dacrabazine	4,882	4,973
#5	Chemotherapy	1,334,932	1,369,176
#6	Cost effectiveness analysis	46,965	48,803
#7	Cost utility analysis	38,823	39,777
#8	Economic analysis	171,594	171,594
#11	#2 OR #3	466,329	466,329
#12	#4 OR #5	1,335,498	1,369,758
#12	#6 OR #7 OR 8	172,188	181,591
#13	#1 AND #11 AND #12	24	1

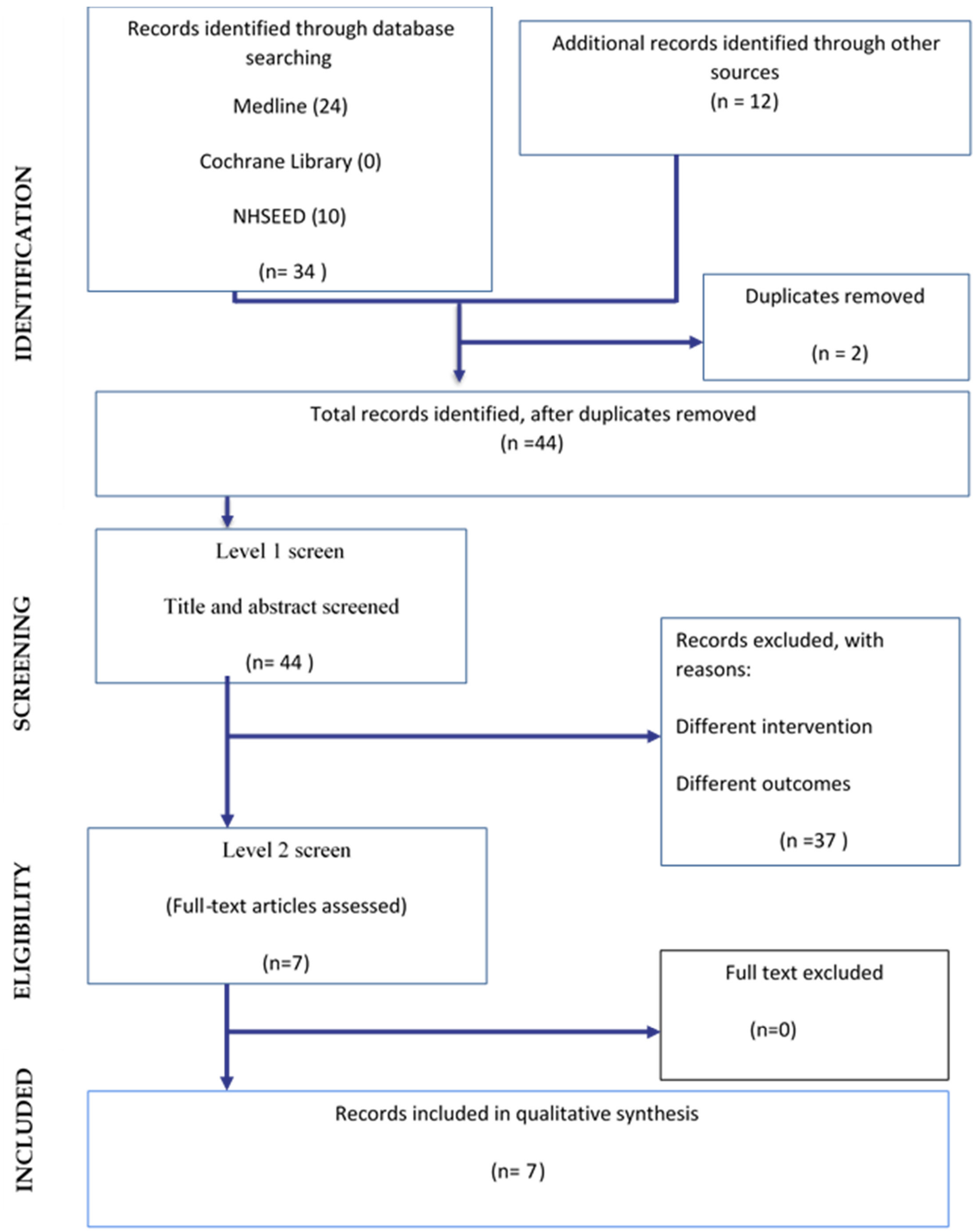

Figure S1: PRISMA flowchart for pharmaco-economic studies

Table S3: Overview of selected original pharmacoeconomic studies

Author, Year, Location	Pike et al, 2017 Norway [3]	Shih et al, 2015 USA [8]	Curl et al, 2014 USA [9]	Guerra et al, 2019 Brazil [10]	Beale et al, 2013 UK[[11] (NICE technology appraisal)
**Intervention**	Ipilimumab<br>Nivolumab<br>Pembrolizumab<br>Dabrafenib<br>Vemurafenib<br>Cobimetinib<br>Trametinib	Vemurafenib<br>Dabrafenib	Vemurafenib<br>Vemurafenib followed by ipilimumab	Monotherapy: Vemurafenib or dabrafenib<br><br>Combination of vemurafenib + cobimetinib or dabrafenib+trametinib	Vemurafenib
**Comparator**	Dacarbazine	Dacarbazine	Dacarbazine	Dacarbazine	Dacarbazine
**Perspective**	Public payer	Societal	Societal	Public payer	National healthcare system
**Time horizon**	10 years	Lifetime	Lifetime	10 years	30 years
**Model type Cycle**	Markov<br>Monthly cycle	Markov<br>Monthly cycle	Decision tree	Markov<br>Monthly cycle	Markov<br>Weekly cycle
**Taux actualisation**	4%	not specified	3%	5%	3.5%
**Utilities**	Vemurafenib<br>PFS: 0.72<br>PD: 0.67	Dacarbazine<br>PFS: 0.69<br>PD: 0.45<br>Vemurafenib<br>PFS: 0.73<br>PD: 0.49	PFS: 0.8<br>PD: 0.52	PFS with dacarbazine: 0.75<br>PFS with vemurafenib: 0.767<br>PD with dacarbazine: 0.677<br>PD with vemurafenib: 0.677	PFS with dacarbazine: 0,767<br>PFS with vemurafenib: 0,806<br>PD with dacarbazine: 0,59<br>PD with vemurafenib: 0,59
**Sponsor**	Norwegian Knowledge Centre for Health Services	Not specified	None	Not specified	Not specified
**ICER vemurafenib versus dacarbazine**	281 932 Euro/QALY	319 972 USD/QALY	353 993 USD/QALY	266 453,83 USD/QALY	94 267,0 £/QALY and 64 891,0 £/LYG<br>Following a managed entry agreement, ICER is reduced to 56 410,0 £/ QALY
**Etude clinique Socle**	BRIM-3	BRIM-3	BRIM-3	BRIM-3	BRIM-3
**Conclusion**	None of the interventions is cost-effective in Norway.<br>81% reduction in the price of vemurafenib would be required to reach an efficiency threshold of 55,850/QALY.	The intervention is not cost-effective at the threshold of 100,000 USD/QALY	The cost-effectiveness threshold of USD 100.000/QALY could be reached with a cut of 72% on the price of vemurafenib.	Targeted therapies are not cost-effective compared with chemotherapy.<br>A price reduction of 85-90% (monthly cost of 581.58 USD) would be required to reach a threshold of 3 times GDP/QALY.	A confidential cut on the price of vemurafenib (through a patient access scheme proposed by the company) allowed to reach the upper limit of the UK's end-of-life cost effectiveness threshold.

Table S4: Overview of systematic reviews of pharmaco-economic studies

Publication	Objective	Funding	Conclusion
Gorry C et al.,2020 [4]	To identify published pharmacoeconomic studies of treatments for advanced melanoma and evaluate their methodological quality, hypotheses, and results as a preliminary step to preparing a health technology assessment report.	The authors reported that they have not received any funding for conducting this systematic review.	Although the results in terms of costs and QALYs gained vary for the different treatments, the results of the different studies concur that BRAF monotherapies and combinations are not cost-effective at the proposed prices compared to chemotherapy in any jurisdiction.
Rubio-Rodriguez D et al., 2017 [12]	To identify and discuss the results of published pharmaco-economic studies investigating the cost-effectiveness of treatments for advanced melanoma.	The authors reported that they have not received any funding for the conduct of this systematic review.	Although the QALYs generated by BRAF inhibitors were higher than those generated by chemotherapy in the various studies, the treatments were not considered cost-effective in any country.

Table S5: Search strategy for clinical inputs

**Databases and journals**	
**Medline (via PubMed), Cochrane Library**	
**Timeframe**	
**Date: January 2011 to December 2020**<br>**Update: February 2021**	
**Inclusion criteria**	
**Population**	Patient with unresectable stage III and/or stage IV cutaneous melanoma positive for BRAF V600 mutation
**Intervention**	Vemurafenib
**Comparator**	Dacarbazine
**Outcomes**	Efficacy (Overall survival, Progression Free Survival)
**Study design**	Systematic reviews and meta-analyses
**Exclusion Criteria**	
**Language**	Other than English or French
**Keywords**	
9. Advanced melanoma.<br>10. Vemurafenib<br>11. Zelboraf<br>12. Dacarbazine<br>13. Effectiveness<br>14. Efficacy<br>15. Overall survival<br>16. Progression Free Survival<br>17. Meta analysis	

Table S6: Search results for clinical inputs on PubMed

Search #	Search equation	Number of documents (01/2011-12/2020)	February 2021
#1	Advanced melanoma	7,200	7,740
#2	Vemurafenib	2,433	2,521
#3	Zelboraf	2,433	2,521
#4	Dacarbazine	4,882	4,973
#5	Effectiveness	3,984,493	4,197,223
#6	Efficacy	497,331	531,860
#7	Overall survival	1,099,423	1,165,995
#8	Progression Free Survival	51,558	55,341
#9	Meta analysis	157,878	170,797
#10	#2OR #3	2,433	2,521
#11	#5 OR #6 OR #7 OR #8	4,710,506	4,977,993
#12	#1 AND #4 AND #9 AND #10 AND #11	6	6

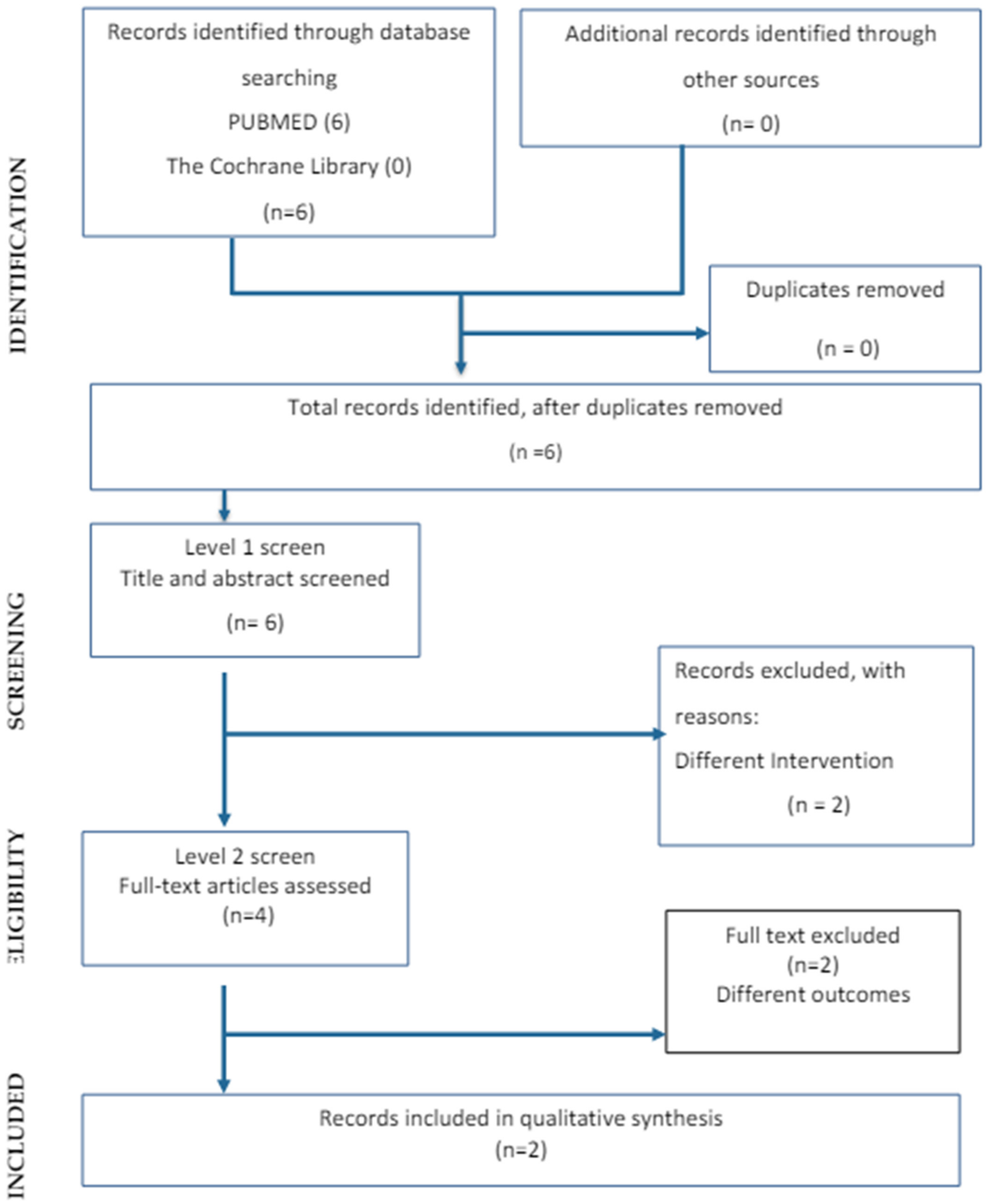

Figure S2: PRISMA flow chart for clinical studies

Table S7: Evidence tables of selected systematic reviews (extracted from FLC)

ABBREVIATED REFERENCE	STUDY	RESEARCH QUESTION	METHOD	RESULTS	CONCLUSIONS	QUALITY OF THE STUDY
Franken et al, 2019 [15]	**Design:** Network meta-analysis (NMA) **Objectives:** A systematic literature review was performed to investigate the relative effectiveness and safety of each systemic treatment option for melanoma. **Location and study period:** The search was performed from January 1, 2010 to March 11, 2019.	**Study population:** Patient with unresectable stage III and/or stage IV cutaneous melanoma. **Intervention:** Systemic treatment for unresectable stage III and/or stage IV cutaneous melanoma. **Comparison:** Network metanalysis **Results analyzed:** Progression-free survival Overall survival	**Type of studies included:** RCTs **Quality of studies:** The quality of the studies was assessed by means of the Cochrane collaboration's tool for assessing risk of bias in randomised trials	**Results:** The SLR identified 28 phase III RCTs involving 14,376 patients. Nineteen and seventeen treatments were included in the effectiveness and safety NMA, respectively. For PFS, dabrafenib plus trametinib (HR PFS: 0.21) and vemurafenib plus cobimetinib (HR PFS: 0.22) were identified as most favourable treatments. Both had, however, less favourable safety profiles. Five other treatments closely followed (dabrafenib [HR PFS: 0.30], nivolumab plus ipilimumab [HR PFS: 0.34], vemurafenib [HR PFS: 0.38], nivolumab [HR PFS: 0.42] and pembrolizumab [HR PFS: 0.46]). In contrast, for OS, nivolumab plus ipilimumab (HR OS: 0.39), nivolumab (HR OS: 0.46) and pembrolizumab (HR OS: 0.50) were more favourable than dabrafenib plus trametinib (HR OS: 0.55) and vemurafenib plus cobimetinib (HR OS: 0.57).	Our NMA identified the most effective treatment options for advanced melanoma and provided valuable insights into each" novel treatment's relative effectiveness and safety. This information may facilitate evidence-based decision-making and may support the optimisation of treatment and outcomes in everyday clinical practice.	High

Pike E, et al. 2017 [3]	**Design:**<br>A systematic review of effectiveness of advanced melanoma treatments<br><br>**Objectives:**<br>To assess the relative effectiveness of seven new drugs (cobimetinib, dabrafenib, ipilimumab, nivolumab, pembrolizumab, trametinib and vemurafenib) used for treatment of patients with advanced malignant melanoma in the Norwegian setting<br>**Location and study period:**<br>Norwegian setting<br>February 2015	**Study population:**<br>Patients with inoperable or metastatic malignant melanoma aged 18 or older<br><br>**Intervention:**<br>Ipilimumab, pembrolizumab, nivolumab, cobimetinib, vemurafenib trametinib and dabrafenib given as monotherapy or in combination with each other<br><br>**Comparison:**<br>Any drug or placebo<br><br>**Results analyzed:**<br>Overall survival (or time to death), Progression free survival (PFS)<br>Heath related quality of life, serious adverse events	**Type of studies included:**<br>Randomized controlled trials<br><br>**Quality of studies:** It was assessed by two independent reviewers using Håndbok for Nasjonalt kunnskapssenter for helsetjenesten.<br><br>Disagreements were resolved by discussions or, if required, by consulting one of the other review authors	**No. studies included:**<br>17 RCTs<br>A total of 7482 patients were included in the 17 trials with a range from 59 to 945 patients in each trial<br><br>**Results:**<br>Ipilimumab: OS 0.69 (0.44 to 1.26), PFS 0.84 (0.54 to 1.52)<br>Dabrafenib: OS 0.73 (0.49 to 1.10), PFS 0.37 (0.22 to 0.63)<br>Nivolumab: OS 0.45 (0.30 to 0.71), PFS 0.50 (0.36 to 0.82)<br>Pembrolizumab: OS 0.46 (0.26 to 0.99), PFS 0.47 (0.30 to 0.76)<br>Trametinib: OS 0.78 (0.49 to 1.22), PFS 0.45 (0.25 to 0.82)<br>Vemurafenib: OS 0.77 (0.54 to 1.10), PFS 0.38 (0.24 to 0.62)	Monotherapies with a PD-1 immune-checkpoint-inhibitor had a higher probability of good performance for OS than monotherapies with ipilimumab or BRAF/MEK inhibitors. The combination treatments had all similar levels of effectiveness to the PD-1 immune-checkpoint-inhibitors.	High

Table S8: Search strategy for utilities in patients with advanced melanoma

Databases and journals	
**Medline (via PubMed), web of science**	
**Timeframe**	
**Date: January 2011 to December 2020**<br>**Update: February 2021**	
**Inclusion criteria**	
We included only systematic reviews, written in English or French, reporting health-related quality of life in the adult advanced melanoma population treated with chemotherapy (dacarbazine) or targeted therapy using direct or indirect methods.	
**Exclusion Criteria**	
**Language**	Other than English or French
**Keywords**	
**1.** Advanced melanoma<br>2. Vemurafenib<br>3. Zelboraf<br>4. Targeted therapy<br>5. Dacarbazine<br>6. Chemotherapy<br>7. Quality adjusted life years<br>8. Preference based quality of life<br>9. Utility<br>10. QALY	

Table S9: Evidence table of the selected studies on health-related quality of life (extracted from FLC)

ABBREVIATED REFERENCE	STUDY	RESEARCH QUESTION	METHOD	RESULTS	CONCLUSIONS	QUALITY OF THE STUDY
Tran et al, 2018 [17]	**Design:** Systematic review and meta-analysis<br><br>**Objectives:** To provide pooled estimates of utilities for people with stage I/II, III or IV melanoma as input for economic evaluations.<br><br>**Location and study period:**<br><br>Australia<br><br>The database searches were completed on May 15, 2017.	**Study population:**<br><br>Patients with advanced melanoma	**Type of studies included:**<br><br>Studies were included if their population comprised patients with melanoma of any stage. There was no restriction on age or other participant characteristics. Studies were included if they either reported utilities directly, or if utilities could be calculated from commonly used health related quality of life surveys including SF-36, SF-12, FACT-M, FACT-G, and QLQ-C30.<br><br>**Quality of studies:** ROBINS-I checklist	**No. studies included:**33 studies reporting 213 utilities.<br><br>**Results:** From meta-analyses, the mean utility for stage I/II melanoma was 0,97 [95% confidence interval (CI) 0,90–0,98]; for stage III melanoma it was 0,77 (95% CI 0,70–0,83); for stage III/IV 0,76 (95% CI 0,76–0,77); and for stage IV melanoma 0,76 (95% CI 0,71–0,81). The difference in utility between stage III and stage IV was not statistically significant (P = 052). For patients with stage I/II, the utility estimate at the time of surgery was 0,77 (95% CI 0,75–0,79), and at 3–12 months post-surgery it was 0,85 (95% CI 084–086). Utility estimates for patients with stage IV melanoma were 0,65 (95% CI 062–069) during the first 3 months of treatment	These robust, evidence-based estimates of health state utilities can be used in economic evaluations of new treatments for patients with early-stage or advanced-stage melanoma.	High

ABBREVIATED REFERENCE	STUDY	RESEARCH QUESTION	METHOD	RESULTS	CONCLUSIONS	QUALITY OF THE STUDY
				and 0,83 (95% CI 081–086) at 4-12 months on treatment. For patients with stage IV melanoma treated with chemotherapy, the utility estimate was 0,52 (95% CI 0,51–0,52), while for those treated with targeted therapy it was 0,83 (95% CI 082–085).		

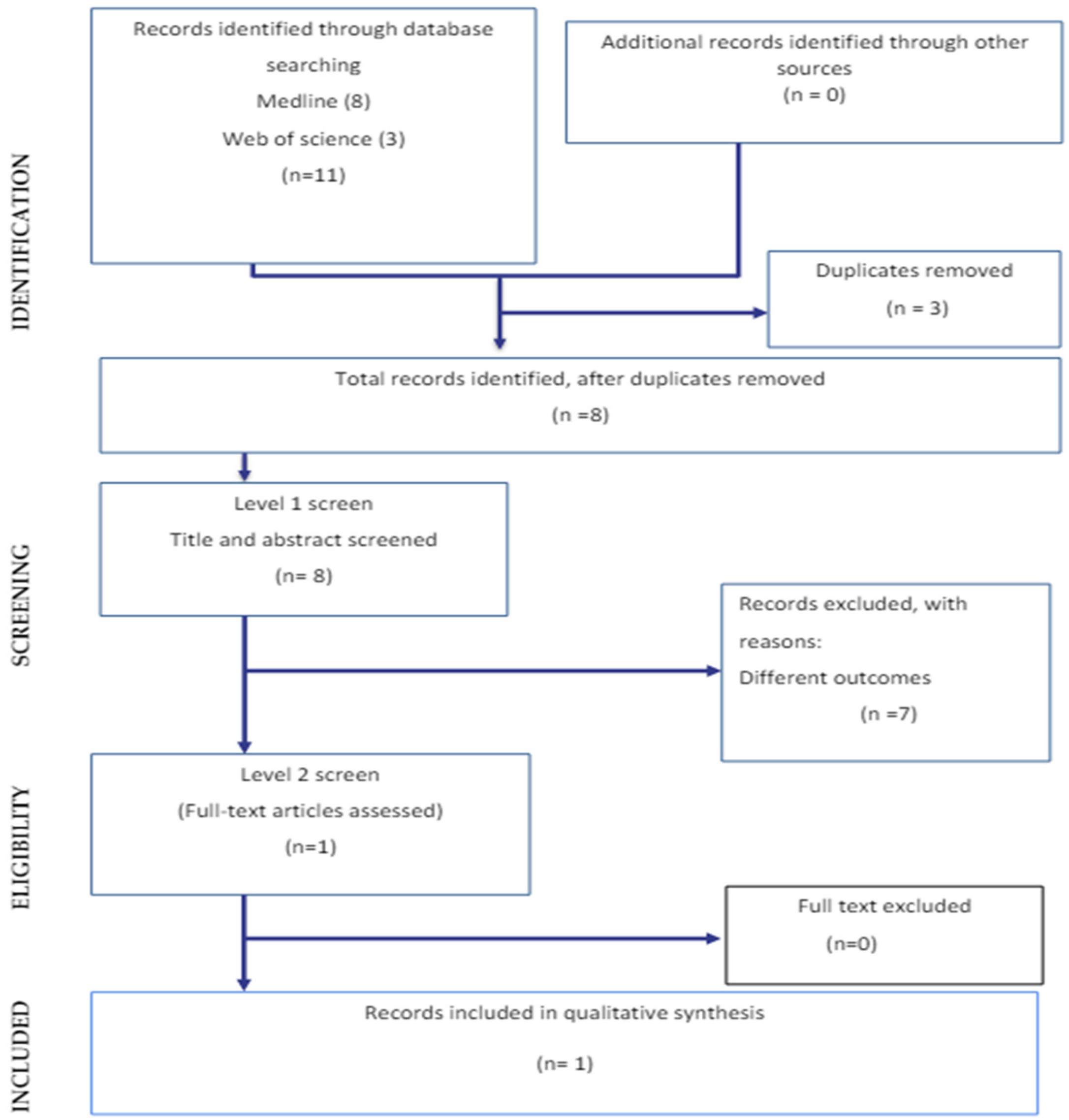

Figure S3: PRISMA flow chart for studies on health-related quality of life
